# Cytotoxicity of Frutalin on Distinct Cancer Cells Is Independent of Its Glycosylation

**DOI:** 10.3390/molecules26164712

**Published:** 2021-08-04

**Authors:** Carla Oliveira, Ana Isabel Freitas, Nair Campos, Lucília Saraiva, Lucília Domingues

**Affiliations:** 1 CBQF—Centro de Biotecnologia e Química Fina—Laboratório Associado, Universidade Católica Portuguesa, Escola Superior de Biotecnologia, Rua Diogo Botelho 1327, 4169-005 Porto, Portugal; cmoliveira@porto.ucp.pt; 2 CEB—Centre of Biological Engineering, University of Minho, 4710-057 Braga, Portugal; ana.isabel.freitas@ceb.uminho.pt; 3 LAQV/REQUIMTE, Laboratόrio de Microbiologia, Departamento de Ciências Biolόgicas, Faculdade de Farmácia, Universidade do Porto, 4050-313 Porto, Portugal; naircampos@gmail.com (N.C.); lucilia.saraiva@ff.up.pt (L.S.)

**Keywords:** recombinant frutalin, isoforms, *Escherichia coli*, targeted anticancer therapy, p53

## Abstract

Frutalin is a plant lectin with beneficial immunobiological action, although the access to its active form is still restricted. Moreover, there is a knowledge gap on isoform activity and glycosylation impact on its bioactivity, and recombinant production protocols were seen as ineffective. Here, a simpler and faster production and purification protocol was developed, attaining a yield of purified frutalin 3.3-fold higher than that obtained previously. Hemagglutination assays confirmed that this frutalin isoform could not agglutinate rabbit erythrocytes, while maintaining the native tetrameric structure, as indicated by DLS analysis, and strong interaction with methyl-alpha-galactose, in fluorescence spectroscopy studies. The cytotoxicity of the recombinant frutalin isoform was shown in a broad panel of human cancer cells: colon (HCT116), melanoma (A375), triple-negative breast cancer (MDA-MB-231), and ovarian (IGROV-1). Treatment with 8.5–11.8 μM TrxFTL reduced proliferation of all cancer cells to half in 48 h. This anti-proliferative effect encompasses the p53 pathway since it was significantly reduced in p53-null colon cancer cells (HCT116 p53^−/−^; GI_50_ of 25.0 ± 3.0 μM), when compared to the isogenic p53-positive cells (HCT116 p53^+/+^; GI_50_ of 8.7 ± 1.8 μM; *p* < 0.002). This recombinantly produced frutalin isoform has relevant cytotoxic effect and its biological activity is not dependent on glycosylation. The developed *E. coli* production and purification protocol generates high yield of non-glycosylated frutalin isoform with potent cytotoxic activity, enabling the development of novel anticancer p53-targeting therapies.

## 1. Introduction

Frutalin lectin has been extensively studied in recent years, mainly in immunobiological research, due to its outstanding biological properties. These include, for example, gastroprotection [1], tissue repair, regeneration and chronic wound healing [2], antidepressant-like effect [3], and antitumor activity, resulting from the recognition of cancer-associated oligosaccharides [4]. As other lectins, frutalin isolated from its natural source (breadfruit seeds) is a mixture of isoforms [5]. Slight (but important) differences in amino acids sequences (from 3 to 7%) were identified in frutalin isoforms [6]. Such differences also account for a partial glycosylation in final samples. It is well-known that each lectin isoform can interact differently with cells, leading to distinct cytoagglutinatination and cytotoxic activities [5]. It is still unclear the role of glycosylation on such biological diversity, while it seems a critical factor on lectins stability [7]. Sample heterogeneity, together with geographical and seasonal source-dependence, and low purification yield, restricts further exploitation of the biomedical properties of frutalin. The bacteria *Escherichia coli* was tested as recombinant host by employing direct cDNA cloning and no fusion partners, but the production yield of soluble frutalin was low [6]. Still, recombinant frutalin revealed agglutination properties and carbohydrate-binding specificity similar to the native lectin [6]. On the other hand, the yeast *Pichia pastoris* has proven to be an effective alternative to obtain soluble, stable, and functional frutalin samples [8]. Interestingly, while native frutalin demonstrated a strong hemagglutinating activity, the recombinant frutalin did not present such capacity, which could either be due to hampering effects of yeast glycosylation or to an intrinsic characteristic of the cloned isoform [8]. Still, recombinant frutalin showed irreversible antiproliferative effect on HeLa cervical cancer cells, as native frutalin, and higher specificity as a biomarker of human prostate cancer [4,9]. Nonetheless, the production and purification process of frutalin in yeast was relatively slow, laborious, and resulted in modest yields [8].

With the great advances in fusion tag technology, for enhancing protein productivity and solubility, mainly in *E. coli*, several difficult-to-express proteins can now be produced in this host at reasonable yields [10,11,12,13]. As such, the aim of this study was to develop a novel strategy to produce and purify higher amounts of biologically active frutalin in *E. coli*, based on previously reported guidelines [11,12,13,14], and to study its antitumor activity. The molecular cloning approach employed in this work consisted in codon bias optimization, fusion with a solubility enhancer (TrxA) and a purification tag (6xHistag), followed by optimization of the culture conditions, and partners’ cleavage. The target frutalin isoform was the one previously produced in *P. pastoris* [8]. Biological activity of the non-glycosylated frutalin obtained in *E. coli* was studied in vitro with rabbit erythrocytes and human cancer cells to ascertain its functionality and elucidate glycosylation importance on cells recognition. In addition, taking advantage of the improved production and purification of this recombinant frutalin isoform, its effect on different human cancer cell lines was for the first time evaluated.

## 2. Results

### 2.1. Expression and Purification of Recombinant Frutalin

**Figure 1:**
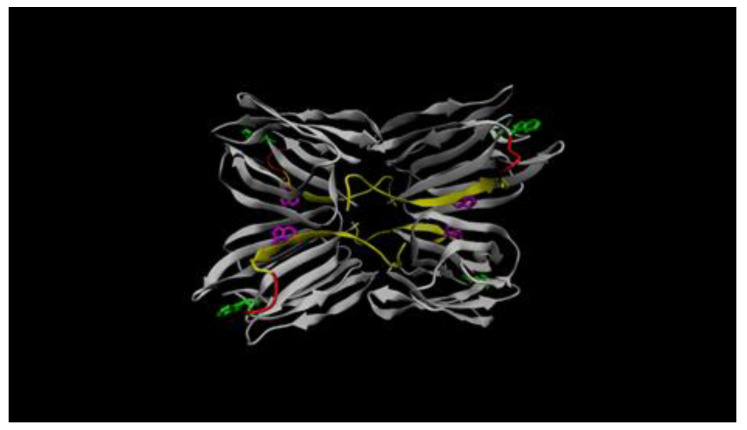
Putative molecular model of recombinant frutalin. The β chains are colored in yellow and the α chains in silver in tetrameric structure. The four amino acid linker TSSN, which binds α and β chains, is represented in red. The carbohydrate-binding site involves the N-terminus of the α chain and is formed by four key residues: Gly25, Tyr146, Trp147, and Asp149 [15]. Alterations in the spatial position of Trp residues upon frutalin binding to carbohydrates, principally in α chain, contribute to intrinsic fluorescence changes. The Trp residues are represented in green and magenta in α and β chains, respectively.

Previously, the frutalin isoform of this work was successfully produced in *P. pastoris* by a 5-days induction process and purified by size exclusion chromatography (SEC) [8]. However, SEC led to a large dilution of recombinant frutalin during separation, while a concentration step was required for downstream applications. Hence, production of frutalin in yeast was found to be a time-consuming process of moderate yield (up to 20 milligrams per liter of culture), whilst the pure product consisted in a mixture of glycosylated and non-glycosylated forms [8]. Then, *E. coli* was employed as alternative host, but resulted in low soluble amounts of protein, despite optimization of codon usage and induction conditions (inducer concentration, temperature, and time of induction) (unpublished results). Furthermore, purification of soluble protein fraction by IMAC was very inefficient. Similar failure was found in the purification of frutalin produced in *P. pastoris* also by IMAC (unpublished results). The difficulty in purifying frutalin in these works using the small 6xHistag placed directly at its N-terminal or C-terminal, in bacteria and yeast, respectively, can be attributed to the location of these ends in the 3D frutalin structure, which according to the built model, are hidden (Figure 1). As it can be observed in the model, the C-terminal of α chains (silver) and the N-terminal of β-chains (yellow) are placed inside the frutalin tetrameric structure (Figure 1), which presents the typical symmetric β-prism fold of jacalin-related lectins [15]. Importantly, in contrast to native frutalin, the first amino acid in α chain (Gly25) is not free in recombinant frutalin, but it is connected to β chain by the linker TSSN (in red; Figure 1), being a key factor in the structural arrangement of the residues involved in the sugar-binding site of d-galactose (Gly25, Tyr146, Trp147, and Asp149) [15]. Not relevant for the model, but determinant for purification, the 6xHistag was placed at the N-terminal of frutalin (β chain) via connection of 13 extra amino acids (which includes the TEV recognition site) (Table 1). These amino acids functioned as a spacing linker that improved the availability of 6xHistag to interact with the Ni^2+^ resin [11], thus enabling the efficiency of frutalin purification by IMAC.

**Figure 2:**
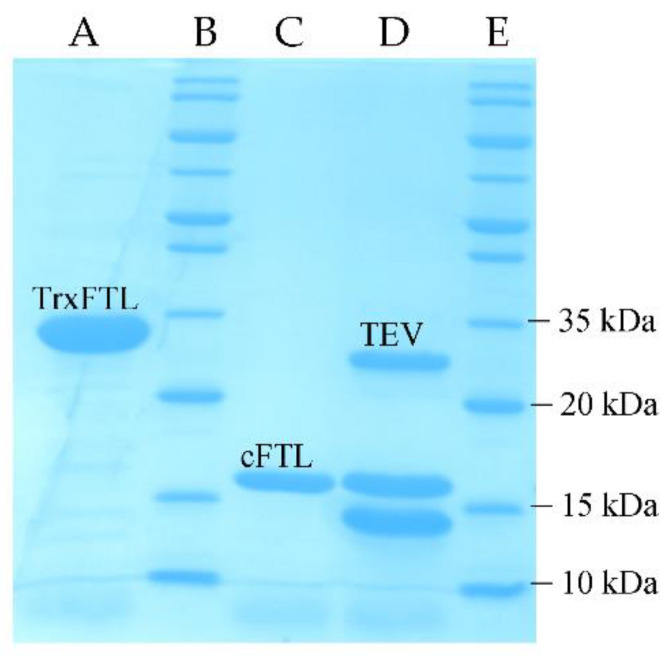
SDS-PAGE analysis of purification of TrxFTL and cFTL from *E. coli*. Lane A, FTL in fusion with TrxA (TrxFTL) purified by IMAC; Lanes B and E, molecular weight marker; Lane C, FTL cleaved and purified by reverse IMAC from TrxFTL; Lane D, products of digestion of TrxFTL with TEV protease (cFTL~17.4 kDa; TrxA~14.3 kDa).

Later, in a comparative tags study, the soluble production of another frutalin isoform was considerably improved by using different solubility enhancers, such as the TrxA, and the 6xHistag for purification by IMAC, being the protein soluble after partners’ removal by TEV cleavage (Tobacco Etch Virus protease) [14]. Thus, a similar strategy was followed with the isoform selected for this work. Indeed, frutalin was produced in soluble form from *E. coli* BL21 in fusion with TrxA (TrxFTL) at high amounts. The fusion protein strategy boosted the availability of FTL by increasing its yield from few micrograms to dozens of milligrams per liter of *E. coli* culture, whilst simplifying the whole production and purification protocol. Roughly, 18 mg of purified TrxFTL per gram of fresh biomass was obtained. IMAC revealed to be a simpler, easier, and quicker procedure than SEC for frutalin purification. Purified TrxFTL migrated in SDS-PAGE (Figure 2), as a homogeneous single band with a molecular weight close to its calculated molecular weight (MW) (~32 kDa; Table 1). Purified cFTL could be observed in gel as a single band of ~17 kDa (Figure 2), which is also consistent with the predicted MW of the cloned frutalin sequence (Table 1). The same pattern in SDS-PAGE gel was also obtained by other frutalin isoform produced and purified in *E. coli*; however, at the μg per liter scale [6]. Thus, it can be concluded that fusion tag technology, together with optimization of codon usage and production and purification process, was essential for obtaining high pure yield of the difficult-to-express frutalin isoform.

### 2.2. Protein Homogeneity Analysis by Dynamic Light Scattering (DLS)

Protein homogeneity was studied by DLS using the intensity distribution method. In this method, high MW aggregates will disproportionately scatter more light in relation to smaller molecules, enabling detection even if present at a relatively low concentration [11]. Distribution is plotted against an apparent hydrodynamic radius, i.e., the radius of a hypothetical sphere that diffuses at the same rate as the particle under study, which is used to estimate the MW of the target molecule using the instrument software, and vice-versa [11].

**Figure 3:**
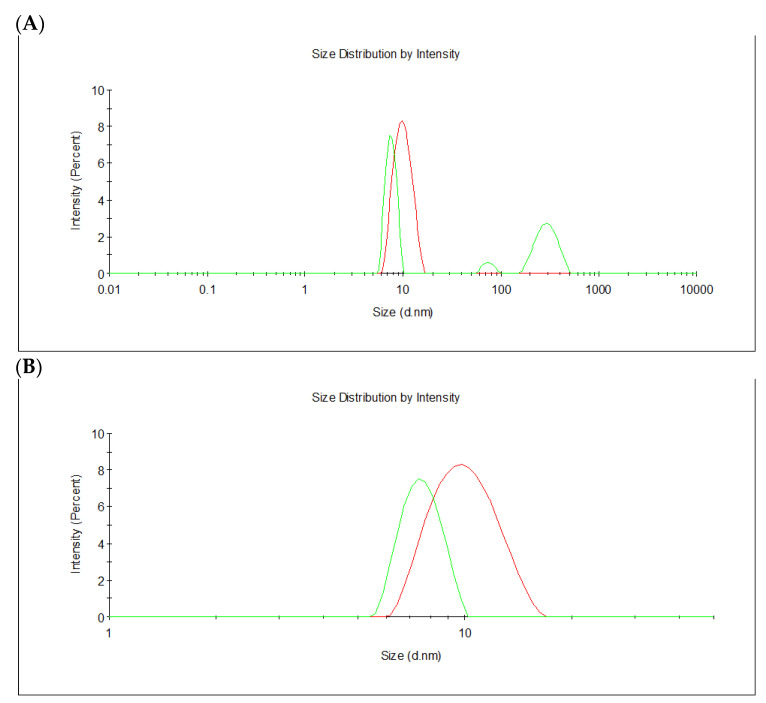
Size distribution by intensity for TrxFTL (red line) and FTL cleaved from TrxFTL (green line). (**A**) Size scale 0.01–10,000 nm diameter; (**B**) size scale 1–50 nm diameter. Estimated diameter for TrxFTL (9.4 nm) is higher than that estimated for cFTL (7.3 nm). Each curve represents the average of 10 independent measurements.

Frutalin is a tetrameric protein in nature [8,16]. If the recombinant frutalin obtained from *E. coli* acquires a tetrameric structure, the expected theoretical MW of TrxFTL would be 126 kDa (monomer with 31.6 kDa; Table 1) and the theoretical MW of cleaved FTL from TrxFTL would be 70 kDa (monomer with 17.4 kDa; Table 1). According to calculations of DLS software, apparent diameter of TrxFTL should be higher than that of cFTL, namely: 9.4 nm (4.7 nm of apparent radius) for TrxFTL and 7.3 nm (3.7 nm of apparent radius) for cFTL. These theoretical measurements are in complete agreement with what can be observed in Figure 3, where the population of TrxFTL and cFTL presents a diameter close to 7.5 nm and 9.5 nm, respectively. Thus, DLS not only suggests tetrameric forms, for either TrxFTL or cFTL, but also that cFTL was perfectly cleaved form TrxFTL. Finally, TrxFTL appears to be highly homogenous, while cFTL seems to have some heterogeneity, due to the presence of high MW aggregates, as indicated by the presence of populations with high diameters (Figure 3A). The high homogeneity in TrxFTL samples enabled its purification without a SEC refining step.

### 2.3. Hemagglutinating Activity

**Figure 4:**
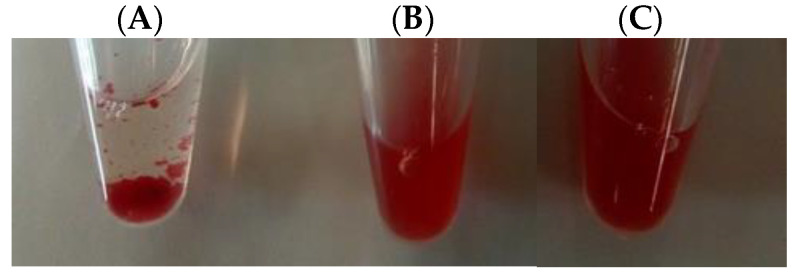
Photos of hemagglutinating assays carried out in 1.5 mL Eppendorfs with native frutalin (**A**), TrxFTL (**B**), and cFTL (**C**) for the higher concentration tested (0.2 mg/mL). A coagulum at the bottom of the Eppendorf is clearly visible in the presence of native frutalin (**A**), but not in the presence of TrxFTL (**B**) and cFTL (**C**).

Native frutalin is known by its strong hemagglutinating activity towards rabbit erythrocytes [8]. Contrarily to native frutalin, no visible hemagglutinating activity was detected in these cells with purified TrxFTL or cFTL (Figure 4). A lectin obtained by recombinant means can lack hemagglutinating activity for basically three reasons: the lectin does not adopt the correct oligomeric conformation (hemagglutination activity presupposes at least two binding sites, e.g., by dimer formation); glycosylation, or other post-translational modification, in case of a lectin produced from a eukaryotic organism, exerts structural effects that can hamper this activity; and the cloned gene corresponds to a isoform that does not have this activity (many lectins are a mixture of isoforms with few amino acids differences, enough for giving them different biological activities). Previously, it was hypothesized that glycosylation might inhibit hemagglutinating activity of frutalin. However, the same frutalin version (same amino acids sequence) produced in a deglycosylated form in *E. coli* (this work) and in a partly-glycosylated form in *P. pastoris* (possibly, at Asn74) [8] did not present this activity, while another isoform with 92% of sequence identity obtained in *E. coli* was able to agglutinate rabbit erythrocytes [6]. Structural studies indicated tetrameric structure for all frutalin versions, including TrxFTL (this work). The isoform having agglutination capacity, while being non-glycosylated, differs in 12 amino acids in a total of 157 [6]. Thus, it can be assumed that the absence of hemagglutinating capacity in frutalin is dependent on the isoform (amino acid sequence), and not on the glycosylation. It is now clear that frutalin isoforms have different biological activities, regardless the presence or absence of glycosylation. Interestingly, the same conclusion about glycosylation was recently taken in the case of other lectins [7,17]. For example, the role of glycosylation of *Curcuma longa* rhizome lectin was studied by structural and activity assays conducted with wild-type lectin, deglycosylated form produced in *E. coli*, glycosylated form produced in *P. pastoris*, and glycosylated mutants N66Q and N110Q, also produced in *P. pastoris*. Circular dichroism, fluorescence spectroscopy, and hemagglutinating studies showed no differences in secondary or tertiary structures, or sugar binding properties, between native lectin and each recombinant lectin form under physiological pH [7]. Although glycosylation was found important to maintain correct lectin folding at acidic pH [7].

### 2.4. Interaction with Methyl-α-Galactose

**Figure 5:**
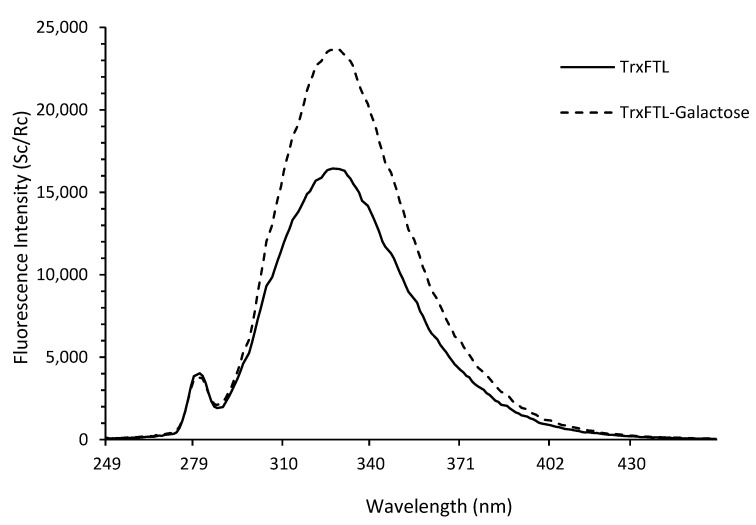
Fluorescence emission spectra of TrxFTL in the absence (solid line) and presence (dashed line) of methyl-α-galactose in PBS.

The fluorescence emission spectrum of TrxFTL showed an emission maximum at 328 nm. The same isoform produced in *P. pastoris* presented a fluorescence emission maximum at 333 nm [8]. Among many sugars studied, the recombinant frutalin produced in yeast demonstrated preference for methyl-α-galactose, which led to high enhancements in frutalin fluorescence emission (~35%) [8]. Thus, the response of fluorescence emission of TrxFTL in presence of this sugar, at the same concentration (100 mM), was analyzed. Similarly, a large increase (~46%) in the maximum fluorescence emission of TrxFTL was observed in the presence of methyl-α-galactose (Figure 5). This result confirms that TrxFTL also binds to this sugar. No interaction with d-galactose was detected. Most probably, TrxFTL has identical sugar affinity to recombinant frutalin produced in yeast. According to previous studies, recombinant frutalin and jacalin produced in *E. coli* presented a ~100-fold lower affinity for methyl-α-galactose (association constants in the order of 10^2^ M^−1^) than the corresponding native lectins (association constants in the order of 10^4^ M^−1^). This is due to the absence of a proteolytic event in the recombinant host related to the cleavage of the four amino acid peptide “T-S-S-N”, which connects the two polypeptides (alpha- and beta-chain) that comprise the lectin [8,18]. Peptide excision presumably reduces the rigidity of frutalin carbohydrate-binding site, increasing the number of interactions with ligands and resulting in multiple-binding sites and anomeric recognition of α-d-galactose sugar moieties [15]. Nevertheless, the loss in sugar binding affinity did not compromise the ability of jacalin or frutalin to recognize cancer cells [4,9,19].

### 2.5. Antitumor Activity

**Figure 6:**
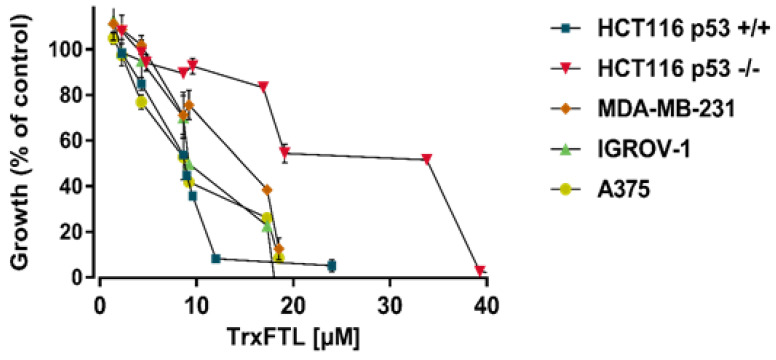
Dose-response curves of the growth inhibitory effect of TrxFTL on human cancer cell lines, determined by the SRB assay, after 48 h treatment. Data are shown as mean ± SEM (*n* = 4–6 independent experiments).

The growth inhibitory effect of TrxFTL and cFTL was initially compared in the human cancer cell line HCT116 p53^+/+^. An identical dose-response curve was obtained for the two recombinant forms, showing once more that the TrxA tag does not interfere with frutalin activity, in agreement with the DLS and hemagglutination assays. Then, TrxFTL was further evaluated in a panel of distinct human cancer cell lines of colon (HCT116 p53^+/+^ and HCT116 p53^−/−^), melanoma (A375), triple-negative breast cancer (MDA-MB-231), and ovarian (IGROV-1), using the SRB assay. A dose-response curve was obtained for TrxFTL in the distinct cancer cells and the GI_50_ was determined after 48 h treatment (Table 2; Figure 6). TrxFTL inhibited the growth of HCT116, A375, IGROV-1, and MDA-MB-231 cells. Interestingly, this anti-proliferative effect of TrxFTL revealed to involve the p53 pathway since it was significantly reduced in p53-null HCT116 cells (HCT116 p53^−/−^), when compared to the isogenic HCT116 p53^+/+^ cells (Table 2). It should also be noted that the effectiveness of TrxFTL on MDA-MB-231 cells, expressing mutant p53, might indicate its ability to target both wild type and mutant p53 forms. In the non-tumor colon cancer cell line CCD-18Co, the GI_50_ value was approximately 2-fold higher than that obtained in the HCT116 p53^+/+^ (16.5 ± 2.2 μM, *n* = 4 independent experiments), which revealed some selectivity of TrxFTL for the tumor cells of the colonic tissue.

## 3. Discussion

In this work, a frutalin isoform with anticancer activity was produced in *E. coli* in fusion with TrxA, for enhancing its solubility and yield, and His6, for facilitating its purification. TrxFTL appeared in SDS-PAGE gel as a single band of ~32 kDa, whereas cleaved FTL migrated in gel as a single band of ~17 kDa (Figure 1), which is in complete agreement with MW of previous recombinant frutalin either expressed in bacteria or yeast hosts [6,8]. As expected, a good yield of purified protein was obtained, 66 mg of protein per liter of culture, which was exceptionally higher (868-fold increase) than the first yield obtained in *E. coli*, 76 μg of pure frutalin per liter of *E. coli* culture [6]. When a microbial host of higher production capacity was employed, *P. pastoris*, the same frutalin isoform could be obtained at the maximum of 20 mg per liter of culture [8]. This means that a 3.3-fold improvement in frutalin yield was achieved in this work. Furthermore, the production and purification protocols were much simplified: shake-flask production was reduced from 5-days to 1-day, and purification, although still consisting in two steps (affinity chromatography plus buffer exchange), was faster and less prone to losses than previous method (size-exclusion chromatography plus concentration by ultrafiltration) [8]. However, one limitation of using the *E. coli* expression system could have been the lack of proper glycosylation, since frutalin is a partly glycosylated lectin in nature. For this reason, the biological activity of TrxFTL was investigated in detail. Unlike other frutalin isoform [6], and native frutalin [18], TrxFTL and cFTL did not present hemagglutinating activity (Figure 4). This result cannot be attributed to conformation issues since the native tetrameric frutalin structure was suggested to be present in TrxFTL and cFTL by DLS analysis (Figure 3). This result explains that the lack of hemagglutination activity in the same tetrameric isoform obtained in yeast is not due to *Pichia* glycosylation pattern. Then, it can be concluded that different frutalin isoforms have different biological activities, as reported for other lectins that are also comprised by a mixture of isoforms [5]. Other observations indicated that the activity of frutalin recombinantly obtained in bacteria is identical to the one obtained in *Pichia*. TrxFTL strongly interacted with methyl-α-galactose (Figure 5), but not with galactose, like frutalin recombinantly produced in yeast [8]. In addition, it showed antiproliferative activity against cancer cells, either in fusion or cleaved from partners. Results of TrxFTL in HCT116^+/+^ cells are quite close to results obtained with same frutalin isoform produced in *P. pastoris*, which showed a GI_50_ of 8.5 ± 0.6 μM (Table 2). Frutalin obtained in yeast also presented a dose-dependent cytotoxicity on human cervical cancer cells, HeLa cells (GI_50_ ~6 μM), whose results were in accordance with native frutalin effects [4]. Since the two frutalin versions of same isoform, partly glycosylated [8] and non-glycosylated (this work), have identical magnitude of cytotoxicity on human cancer cells, it can be concluded that glycosylation is not involved on this activity. This work is in line with other studies reporting that the biological activities of lectins are independent of glycosylation [5,7,20]. This work also shows, for the first time, the cytotoxic effect of frutalin on melanoma, ovarian and triple-negative breast cancer cell lines, which further reinforces the great potential of recombinant frutalin as an anti-cancer drug (Table 2; Figure 6).

The cytotoxicity of plant lectins has been extensively studied on a diversity of cancer cell lines (for a revision, see e.g., Table 3 in [21]). For example, very recently, the antiproliferative activity of the galactose-binding plant lectin BfL-II, produced and purified from *E. coli*, was evaluated in human breast (MCF-7) and colorectal (HT-29) cancer cells, showing to be stronger on colorectal cancer cells, while at much higher concentration than frutalin [20]. Plant lectins exert their antiproliferative activity by inducing programmed cell death pathways (apoptosis and/or autophagy). Frutalin has been shown to induce cell death on HeLa cells by apoptosis [4]. The possible molecular mechanism by which plant lectins induce tumor cell death by apoptosis involves, at first place, the lectin interaction with sugar binding receptors present on the plasma membrane, followed by internalization through endocytosis [22]. This event has been previously observed in the interaction of frutalin produced in *P. pastoris*, and native frutalin, with HeLa cells [4]. Rapidly (within 1-h incubation), both frutalin versions were completely internalized and detected around and inside HeLa nucleus [4]. Sugar binding receptors of jacalin-related lectins, to which family frutalin belongs, are mostly Tn, sTn, and T antigens [21]. These abnormal O-glycans are expressed on several types of cancer, including colon or breast cancers, and are associated with adverse outcomes and poor prognosis [23]. The structure of many lectins, including jacalin, in complex with T antigens have been previously revealed [24]. Interestingly, strong interaction of native frutalin with T antigen (Galb(1-3)GalNac) was observed in our previous work, but no interaction with this sugar could be detected for frutalin produced in yeast [8]. This result suggests the presence of other frutalin receptors, such as methylated glycans, rather than T antigens. Once inside the cells, the lectins can trigger an apoptotic cell death mostly through a mitochondria-mediated pathway, involving relevant players such as p53 [22]. Accordingly, activation of the p53 pathway has already been reported for certain plant lectins. For example, recently, the bean lectin TBLF has shown to induce apoptosis in colon cancer cells by p-p53(ser46) involvement [25]. Nevertheless, no involvement of jacalin-related lectins with p53 pathway has been described so far. Our work adds a novel and important clue on the mechanism underlying frutalin-induced cancer cell death. In fact, the results herein obtained indicate a potential involvement of the p53 pathway in the antiproliferative activity of TrxFTL. Therefore, in a future work, it would be very interesting to deeply elucidate this potential p53-dependent antitumor activity of frutalin.

In conclusion, fusion tag technology was effective in improving frutalin yield in *E. coli*, not interfering with frutalin bioactivity. Moreover, frutalin was obtained in this host as an active anticancer molecule of broad spectrum, which deserves to be further explored based on its great potential in targeted anticancer therapy.

## 4. Materials and Methods

### 4.1. Construction of Expression Vector

Frutalin mature sequence [8], with codons optimized for recombinant expression in *E. coli*, flanked by *Nco*I and *Xho*I recognition sites at the 5′- and 3′-end, respectively, was synthesized by NZYTech. The frutalin synthetic gene was excised from the carrying plasmid by digestion with *Nco*I and *Xho*I enzymes and ligated to the pETM20 vector (EMBL) in fusion with the N-terminal thioredoxin A (TrxA) and His6 tags (linked to the cloning site by a TEV protease recognition sequence) [14]. The construct was transformed and propagated in chemically competent NZY5a *E. coli* cells (NZYTech). For protein expression, the construct was transformed into the *E. coli* strain NZYBL21 (DE3) (NZYTech).

### 4.2. Recombinant Protein Production and Purification

*E. coli* BL21(DE3) cells harboring the recombinant plasmid were grown overnight at 37 ℃ in 10 mL LB medium containing 100 μg/mL of ampicillin. In the day after, 4 × 250 mL of same medium were inoculated with previous culture (1:1000) and grown to an OD600 nm of 0.5 and recombinant protein expression induced with 0.2 mM IPTG for 16 h at 18 ℃. Cells were recovered by centrifugation (at 4 ℃ for 15 min at 10,000 rpm) and lysed with NZY Bacterial Cell Lysis Buffer (NZYTech) supplemented with 1 mM PMSF, according to the manufacturer’s instructions. Soluble cell-free extracts were collected by centrifugation, filtered (0.45 μM pore size), and loaded on a 5 mL Nickel HisTrap column (GE Healthcare) for recombinant protein purification by immobilized metal ion affinity chromatography (IMAC). Purification was conducted according to the manufacturer’s instructions, using 50 mM Tris pH 8.0, 150 mM NaCl with 20 or 40 mM imidazole as running and washing buffer, respectively, and with 300 mM imidazole for the elution buffer [14].

For TrxA-His6 partner removal, the purified fusion protein was digested with TEV-His6 protease overnight at 4 °C at the ratio of 1:20 (*w*/*w*) in running buffer. Elution buffer was previously exchanged with running buffer using PD10 columns (GE Healthcare). The cleaved frutalin was then purified from the fusion tags and protease through reverse purification by incubation with 0.5 mL of HisPur™ Ni-NTA Resin (Thermo Fisher Scientific), following manufacturer’s instructions as given for the batch purification method, using the above-mentioned buffers, but in which the target protein (cFTL) was collected from the resin washing steps.

Purified proteins were analyzed by SDS-PAGE using 15% (*w/v*) acrylamide gels, followed by BlueSafe staining (NZYTech). Imidazole removal from proteins, and any other buffer exchange, was performed using PD10 columns (GE Healthcare). The concentration of the recombinant proteins was estimated from the absorbance at 280 nm using the respective molar extinction coefficients. Recombinant proteins were maintained at 4 ℃ until their use in subsequent in vitro studies.

### 4.3. Dynamic Light Scattering (DLS)

The size distribution of protein samples was determined with a Malvern Zetasizer, MODEL NANO ZS (Malvern Instruments Limited, Worcestershire, UK). Protein in phosphate buffer (1 mL) at a concentration between 0.8–1.0 mg/mL was analyzed at room temperature using a polystyrene cell. Ten measurements per sample were performed. Protein samples were filtrated through 0.45 μm pore. Zetasizer software tool “MW & Shape Estimates” (Worcestershire, UK) was used to determine expected hydrodynamic radius using theoretical MW of proteins. The online ExPASy ProtParam tool was used to calculate MW of proteins using primary amino acid sequences.

### 4.4. Hemagglutination Assays

Hemagglutinating studies were conducted as previously reported [8]. Briefly, lectins were mixed 1:2 with a rabbit erythrocytes solution (2% (*v*/*v*) in 0.15 M NaCl) in the range 0.002–0.2 mg/mL in Eppendorfs, and incubated at 37 ℃ for 30 min, plus another 30 min at room temperature. Thereafter, samples were visually inspected for the presence or absence of agglutination. Native frutalin, obtained as previously described [8], was included as positive control. Three independent assays were performed.

### 4.5. Recombinant Frutalin Three-Dimensional (3D) Model Building

The fully automated protein homology-modelling server SWISS-MODEL [26] was used to predict and evaluate the 3D model structure of recombinant frutalin. The X-ray crystal structure of the frutalin from Artocarpus incisa (PDB ID: 4WOG) served as template. Molecular illustrations were prepared using VMD [27].

### 4.6. Fluorescence Studies

Fluorescence studies were performed as previously described [8] using Spectrofluorometer Horiba Aqualog 800 (Boeblingen, Germany). Gains in the intrinsic fluorescence emission of recombinant frutalin in the presence of the non-fluorescent sugar methyl-α-galactose (100 mM) were recorded at 328 nm. Data was analyzed using OriginPro 2015 software ((Boeblingen, Germany).

### 4.7. Human Cancer Cell Lines and Growth Conditions

Human colon adenocarcinoma HCT116 cell lines, expressing p53 (HCT116 p53^+/+^) and respective p53 knockout (HCT116 p53^−/−^), were provided by B. Vogelstein (The Johns Hopkins Kimmel Cancer Center, Baltimore, MD, USA); melanoma A375 cell line was purchased from CLS Cell Lines Service GmbH (Eppelheim, Germany); human ovarian carcinoma IGROV-1 was provided by Professor Leonor David from Instituto de Investigação e Inovação em Saúde, i3S (Porto, Portugal), human breast adenocarcinoma MDA-MB-231 and normal colon CCD-18Co cell lines were purchased from ATCC (Manassas, VA, USA). Human cell lines used were routinely cultured in RPMI-1640 medium with UltraGlutamine (Lonza, VWR), excepting CCD-18Co cells that were cultured in EMEM (Lonza, Ingrenor, Porto, Portugal), supplemented with 10% fetal bovine serum (FBS; Gibco, Alfagene, Lisboa, Portugal), and maintained at 37 ℃ with 5% CO_2_. Cells were routinely tested for mycoplasma infection using the MycoAlert™ PLUS mycoplasma detection kit (Lonza).

### 4.8. Sulforhodamine B (SRB) Assay

Human cell lines were seeded in 96-well plates at a density of 5.0 × 10^3^ cells/well (for p53^+/+^ and p53^−/−^ HCT116, IGROV-1, MDA-MB-231, and CCD-18Co cells) and 4.5 × 10^3^ cells/well (for A375 cells) for 24 h. Cells were treated with serial dilutions of TrxFTL for additional 48 h. Recombinant frutalin from *P. pastoris*, obtained as previously described [8], was included as positive control. Effects on cell proliferation were measured by SRB assay, as previously described [28], and the concentration that induced half of growth inhibition (GI_50_) was determined for each cell line using the GraphPad Prism software version 7.0 (La Jolla, CA, USA).

Since the effects of TrxFTL and cFTL on cancer cells were identical, this study was conducted with TrxFTL, to avoid extra purification steps of recombinant frutalin.

### 4.9. Statistical Analysis

Statistical significance was calculated for each data set based on at least three independent experiments. Data were analyzed using GraphPad Prism software v7.0 (San Diego, CA, USA) and are presented as means ± SEM. Unpaired Student’s *t*-test (two-tailed) was applied to each dataset in order to compare average values; *p* < 0.05 was considered statistically significant.

## Figures and Tables

**Table 1:** Amino acid sequences of frutalin produced in *P. pastoris* and *E. coli*, and calculated MW and pI.

Frutalin Version	Amino Acid Sequence	Theoretical pI/Mw	Reference
Frutalin produced in *P. pastoris*	***EF***NQQSGKSQTVIVGPWGAKVS**TSSN**GKAFDDGAFTGIREIN LSYNKETAIGDFQVVYDLNGSPYVGQNHKSFITGFTPVKISLD FPSEYIMEVSGYTGNVSGYVVVRSLTFKTNKKTYGPYGVTSGT PFNLPIENGLIVGFKGSIGYWLDYFSMYLSL *	6.87/17381.52	[8]
Frutalin produced in *E. coli* in fusion with TrxA (TrxFTL)	MSDKIIHLTDDSFDTDVLKADGAILVDFWAEWCGPCKMIAP ILDEIADEYQGKLTVAKLNIDQNPGTAPKYGIRGIPTLLLFKN GEVAATKVGALSKGQLKEFLDANLAGSGSGHM**HHHHHH**S SGENLYFQGA***MG***NQQSGKSQTVIVGPWGAKVS**TSSN**GKAF DDGAFTGIREINLSYNKETAIGDFQVVYDLNGSPYVGQNHKS FITGFTPVKISLDFPSEYIMEVSGYTGNVSGYVVVRSLTFKTNK KTYGPYGVTSGTPFNLPIENGLIVGFKGSIGYWLDYFSMYLSL *	5.97/31672.78	This work
Frutalin produced in *E. coli* cleaved from TrxFTL (cFTL)	GA***MG***NQQSGKSQTVIVGPWGAKVS**TSSN**GKAFDDGAFTGI REINLSYNKETAIGDFQVVYDLNGSPYVGQNHKSFITGFTPV KISLDFPSEYIMEVSGYTGNVSGYVVVRSLTFKTNKKTYGPYG VTSGTPFNLPIENGLIVGFKGSIGYWLDYFSMYLSL *	8.05/17421.61	This work

ENLYFQG: TEV recognition sequence; ***EF*** and ***MG***—amino acids from cloning restriction sites; **TSSN**—linker; **HHHHHH**—6xHistag; *—STOP codon.

**Table 2:** Effect of frutalin produced in *E. coli* in fusion with TrxA (TrxFTL) on the growth of different human cancer cell lines and effect of frutalin produced in *P. pastoris* on the growth of HCT116 p53^+/+^ cell line (control).

Frutalin Version	Cancer Cell Lines	GI_50_ (μM)
Frutalin produced in *P. pastoris*	HCT116 p53^+/+^	8.5 ± 0.6
Frutalin produced in *E. coli* in fusion with TrxA (TrxFTL)	HCT116 p53^+/+^	8.7 ± 2.6
	HCT116 p53^−/−^	25.0 ± 3.0 **
	A375	8.5 ± 0.8
	IGROV-1	10.3 ± 0.7
	MDA-MB-231	11.8 ± 1.1

GI_50_ values were determined by SRB assay after 48 h treatment (growth obtained with vehicle was set as 100%). Data are shown as mean ± SEM of four to six independent experiments; values of HCT116 p53^−/−^ cells significantly different from HCT116 p53^+/+^ cells are indicated (** *p* = 0.002, unpaired Student’s *t*-test).

## Sample Availability

Samples of the compounds are available from the authors at reasonable request.
